# Ultrastructural and Cytotoxic Effects of *Metarhizium robertsii* Infection on *Rhipicephalus microplus* Hemocytes

**DOI:** 10.3389/fphys.2019.00654

**Published:** 2019-05-29

**Authors:** Jéssica Fiorotti, Rubem Figueiredo Sadok Menna-Barreto, Patrícia Silva Gôlo, Caio Junior Balduino Coutinho-Rodrigues, Ricardo Oliveira Barbosa Bitencourt, Diva Denelle Spadacci-Morena, Isabele da Costa Angelo, Vânia Rita Elias Pinheiro Bittencourt

**Affiliations:** ^1^Programa de Pós-Graduação em Ciências Veterinárias, Instituto de Veterinária, Universidade Federal Rural do Rio de Janeiro, Seropédica, Brazil; ^2^Laboratório de Biologia Celular, IOC, Fundação Oswaldo Cruz, Rio de Janeiro, Brazil; ^3^Departamento de Parasitologia Animal, Instituto de Veterinária, Universidade Federal Rural do Rio de Janeiro, Seropédica, Brazil; ^4^Laboratório de Fisiopatologia, Instituto Butantan, São Paulo, Brazil; ^5^Departamento de Epidemiologia e Saúde Pública, Instituto de Veterinária, Universidade Federal Rural do Rio de Janeiro, Seropédica, Brazil

**Keywords:** entomopathogenic fungi, cell death, fungal infection, tick, immunity

## Abstract

*Metarhizium* is an entomopathogenic fungus widely employed in the biological control of arthropods. Hemocytes present in the hemolymph of invertebrates are the cells involved in the immune response of arthropods. Despite this, knowledge about *Rhipicephalus microplus* hemocytes morphological aspects as well as their role in response to the fungal infection is scarce. The present study aimed to analyze the hemocytes of *R. microplus* females after *Metarhizium robertsii* infection, using light and electron microscopy approaches associated with the cytotoxicity evaluation. Five types of hemocytes (prohemocytes, spherulocytes, plasmatocytes, granulocytes, and oenocytoids) were described in the hemolymph of uninfected ticks, while only prohemocytes, granulocytes, and plasmatocytes were observed in fungus-infected tick females. Twenty-four hours after the fungal infection, only granulocytes and plasmatocytes were detected in the transmission electron microscopy analysis. Hemocytes from fungus-infected tick females showed several cytoplasmic vacuoles with different electron densities, and lipid droplets in close contact to low electron density vacuoles, as well as the formation of autophagosomes and subcellular material in different stages of degradation could also be observed. *M. robertsii* propagules were more toxic to tick hemocytes in the highest concentration tested (1.0 × 10^8^ conidia mL^−1^). Interestingly, the lowest fungus concentration did not affect significantly the cell viability. Microanalysis showed that cells granules from fungus-infected and uninfected ticks had similar composition. This study addressed the first report of fungal cytotoxicity analyzing ultrastructural effects on hemocytes of *R. microplus* infected with entomopathogenic fungi. These results open new perspectives for the comprehension of ticks physiology and pathology, allowing the identification of new targets for the biological control.

## Introduction

Ticks are obligate hematophagous ectoparasites relevant to public and veterinary health (De la Fuente et al., 2008; Kernif et al., 2016). The cattle tick, *Rhipicephalus microplus*, has an enormous negative impact on livestock. The economic impact has increased, particularly in tropical countries and financial losses of USD 3.24 billion per year only in Brazil have been reported (Grisi et al., 2014). The improper use of chemical acaricides is frequently reported and causes the raising of resistant tick populations in addition to the environmental, meat, and milk contamination. Accordingly, alternative control methods using entomopathogenic fungi have been studied to decrease the use of these chemicals (Schank and Vainstein, 2010; Camargo et al., 2012; Perinotto et al., 2017).

*Metarhizium anisopliae* sensu lato (s.l.) is a complex of species of cosmopolitan entomopathogenic fungi which includes species that can infect specific hosts, as *Metarhizium acridum* with Orthoptera insects, or a wider range of insect groups, such as *Metarhizium robertsii* and *M. anisopliae* sensu stricto (s.s.). These fungi are also considered endophytes and rhizosphere competent (Hu et al., 2014; Vega, 2018). All *R. microplus* life stages have been shown to be sensitive to entomopathogenic fungal infection (Alonso-Díaz et al., 2007; Leemon and Jonsson, 2008), either *in vitro* assays (Perinotto et al., 2017), associated with chemical acaricides (Bahiense et al., 2008; Webster et al., 2015), or *in vivo* tests using available commercial products (Camargo et al., 2016).

*Metarhizium* infection starts with the fungal propagule attachment on the hosts’ surface and active penetration (Madelin et al., 1967). After penetration, the hyphae present in the hemolymph differentiate into blastospores and the host dies due to a set of occurrences such as: physical damage, pathological changes occurring in the hemolymph, histolytic action, blocking of the digestive system, and even production of micotoxins (Bidochka et al., 1997; Alves, 1998; Roberts and St Leger, 2004). Nevertheless, the host can fight against the fungal infection triggering its immune system by starting processes such as phagocytosis, nodule formation, melanization, encapsulation, and secretion of antimicrobial peptides (Kopácek et al., 2010; Hajdušek et al., 2013).

Tick immune system is composed of humoral and cellular defense responses (Schmidt et al., 2001; Lavine and Strand, 2002). In general, the humoral immune response involves several antimicrobial peptides, reactive oxygen species and enzymatic cascades that regulate coagulation and hemolytic melanization processes (Gillespie et al., 1997; Smith and Pal, 2014). The cellular reactions are triggered immediately after the microorganism invasion and directly involve the attack of these microorganisms by hemocytes (Tan et al., 2013). Hemocytes are circulating cells present in the hemolymph that also be found connected to the fatty body, nephrocytes, and salivary glands (Sterba et al., 2011). Innate immune mechanisms have already been reported for several invertebrates (Brayner et al., 2007; Nation, 2016; Iacovone et al., 2018), including ticks (Gillespie et al., 1997; Johns et al., 2001; Feitosa et al., 2015), considering phagocytosis as the most important innate response (Bayne, 1990; Ehlers et al., 1992), that in ticks is mediated mainly by granulocytes and plasmatocytes (Dolp, 1970; Fujisaki et al., 1975; Kuhn and Haug, 1995; Zhioua et al., 1996; Inoue et al., 2001; Borovicková and Hypsa, 2005; Feitosa et al., 2018). Despite this, little has been reported about cellular immune response in *R. microplus* ticks, since most studies are based on the tick humoral responses (Nakajima et al., 2003; Fogaça et al., 2004; Angelo et al., 2014; Chávez et al., 2017).

Hemocytes are extremely relevant for studies about interactions between the host and its pathogens (Jones, 1962; Crossley, 1975; Lackie, 1980; Gupta, 1986; Paskewitz and Christensen, 1996; Chen and Lu, 2018; Iacovone et al., 2018). Arthropods’ hemocytes classification and terminology were framed mostly based on insect studies, while this literature is still scarce for ticks (Nevermann et al., 1991; Borovicková and Hypsa, 2005; Araújo et al., 2008; Cunha et al., 2009; Laughton et al., 2011). Additionally, compared to insects, literature about the cell response of ticks challenged with pathogens is poorly explored.

Studies reported that circulating hemocytes of adult mosquitoes infected with pathogens may decrease (Hillyer et al., 2005; Castillo et al., 2006, 2011; Brayner et al., 2007; Bryant and Michael, 2014) and have their morphology altered (Au et al., 2003; Kwon et al., 2014). It is known that the number of circulating hemocytes of *R. microplus* ticks infected with entomopathogenic fungi dropped off in comparison to uninfected females (De Paulo et al., 2018), despite this, to the present date, alterations in the morphology of hemocytes from *R. microplus* ticks infected with pathogens have not been reported yet, as studies were conducted mainly with *R. sanguineus* and *Dermacentor variabilis* ticks (Eggenberger et al., 1990; Ceraul et al., 2002; Feitosa et al., 2015, 2018). Undoubtedly, further approaches to understand the immune system of ticks have to overcome the discussion of hemocyte types, especially when its identification is based just on morphological analyzes, while histochemical differentiation and functional aspects are largely neglected. Accordingly, the aim of this study was to analyze the hemocytes of *R. microplus* females infected or not by *M. robertsii*, using light microscopy, cytotoxicity test, transmission electron microscopy (TEM), and transmission electron microscopy with energy-dispersive X-ray spectrometer (TEM-EDS).

## Materials and Methods

### Fungal Isolate and Tick Obtainment

One fungal isolate, *M. robertsii* ARSEF 2575, was used in the present study. The fungal isolate was obtained from the Agriculture Research Service Collection of Entomopathogenic Fungal Cultures (ARSEF) (USDA-US Plant, Soil and Nutrition Laboratory, Ithaca, NY, United States). Cultures were grown on PDA (potato dextrose agar) at 25°C ± 1°C and ≥80 relative humidity (RH) for 14 days. A conidial suspension was used to infect the ticks.

*Rhipicephalus microplus* fully engorged ticks were obtained from artificially infested animals [the present experiment was part of a project approved by the Veterinary Institute Ethics Committee of the Federal Rural University of Rio de Janeiro (CEUA UFRRJ no. 037/2014)]. After collection on the stalls floor, fully engorged tick females were immersed in running water followed by 0.05% sodium hypochlorite solution for three min, then dried, identified and weighed for homogeneous division of the groups.

### Entomopathogenic Fungi Inoculation Into *R. microplus* Ticks

Fungal suspensions were prepared by suspending *M. robertsii* ARSEF 2575 conidia in 0.1% polyoxyethylene sorbitan monooleate (Tween^®^80) sterile aqueous solution (v/v). For fungal inoculation, engorged females were divided into seven homogeneously weighed groups with 20 females each (for the bioassay) and three homogeneously weighed groups with 20 females each (for the microscopy analysis). The negative control group did not receive any treatment (tick females under physiological conditions), and treated females were inoculated with 5 μL of ARSEF 2575 fungal suspension or 5 μL 0.1% Tween^®^80 aqueous solution (v/v) into the foramen located between the dorsal scutum and the capitulum, using an insulin syringe (Johns et al., 1998). All the experiments were performed in triplicate (except the bioassay) and repeated three times.

### Bioassay With Engorged Females

Tick females were homogeneously weighed based on the Yule’s formula (Sampaio, 2002). Groups were inoculated as mentioned before and divided as follows: A group was treated with 5 μL 0.1% Tween^®^80 aqueous solution (v/v) and the fungus-infected groups were infected with six different fungal concentrations (1.0 × 10^3^, 10^4^, 10^5^, 10^6^, 10^7^ or 10^8^ conidia mL^−1^). Fungal suspensions were adjusted to each concentration using Neubauer’s chamber. Fungal concentrations that were used in the present bioassay were the same used in the forward described cell viability test. The highest concentration (1.0 × 10^8^ conidia mL^−1^) was defined based on the concentration commonly used for topical treatments in ticks. Tick females were maintained at 27 ± 1°C and ≥80% RH in the dark. Tick mortality was recorded 24 and 48 h after the treatments.

### Hemolymph Collection

Ticks infected with fungi (each tick infected with 5 μL of 1.0 × 10^7^ conidia mL^−1^, a sublethal concentration), ticks inoculated with Tween^®^80 aqueous solution (v/v) and the negative control group (ticks under physiological conditions, a untreated group) had their hemolymph collected according to Angelo et al. (2010) through the ruptured cuticle at the dorsal surface of the tick female using a needle, 24 h after infection. The hemolymph drops were collected with a capillary glass coupled to a flexible rubber. Samples were placed in microtubes containing 30 μL protease inhibitor cocktail (Inhibit^®^Sigma-Aldrich) and 82 μL saline buffer (1.5 M NaCl, 50 mM EDTA, pH 7.2). Microtubes were kept on ice throughout the collection.

### Cell Viability Test

Three hundred engorged tick females were used for the cell viability test. Hemolymph of uninfected females was collected and the hemocytes harvested according to De Paulo et al. (2018). Hemocytes were resuspended in 50 mL sterilized Leibovitz’s L-15 culture medium (Gibco^®^) at double strength, supplemented with 20% fetal bovine serum (FBS), 2 g. L^−1^ glucose, and 100 UI mL^−1^ penicillin, adjusted to pH 7.0–7.2. Cells were then seeded into 96 well plates at 5 × 10^4^ cells mL^−1^ in a final volume of 170 μL culture medium. Cells were allowed to attach to the well surface for 1 h. Hemocytes were then exposed to 10 μL conidial suspensions using six different concentrations (1.0 × 10^3^, 10^4^, 10^5^, 10^6^, 10^7^, or 10^8^ conidia mL^−1^) chosen to make it possible to assemble a kinetic curve. 24 h after fungal exposure, 20 μL of resazurin (Sigma-Aldrich) at 0.13 mg mL^−1^ were added in each well, and the plate incubated for 1, 2, 3, and 4 h. Fluorescence was read using a microplate reader Chameleon (Hidex^®^) at 530 nm excitation and 590 nm emission. Relative hemocyte viability was calculated considered unexposed cells as controls (≈100% viability). Experiments were carried out in triplicate and repeated three times with new engorged females and a new batch of fungus.

### Light Microscopy Analysis

#### Hemolymph Smear

Drops from total hemolymph (plasma and hemocytes) of untreated ticks (negative control group), ticks inoculated with 0.1% Tween^®^80 and ticks infected with 10^7^ conidia mL^−1^ were collected 24 h after infection and placed directly onto glass slides, dried at room temperature for 20–30 min, and fixed in methanol PA (Sigma-Aldrich) for 3 min. Slides were then stained with Giemsa (Sigma-Aldrich) [diluted 1:9 in buffered distilled water Brayner et al. (2005)] for 30 min, rapidly washed with buffered distilled water, and observed by light microscope. Five stained slides were performed per group. One hundred cells per slide were count for differential hemocytes counts. Differential hemocytes counts were expressed based on the amount of each hemocyte type in the total cells counted.

#### Hemocytes in Historesin

For light microscopy, hemocytes from ticks infected with 10^7^ conidia mL^−1^ (collected 24 h after infection) and hemocytes from Tween-inoculated ticks were removed from plasma (De Paulo et al., 2018) and immediately immersed in 4% paraformaldehyde in 0.2 M Millonig buffer (pH 7. 2) for 6 h at room temperature. Samples were then centrifuged at 500 × *g* for 3 min, supernatant was discarded, the hemocytes pellet dehydrated in ascending ethanol series (70–100%), embedded in pure historesin overnight, and finally embedded in historesin plus hardener (Leica) at 4°C for 24 h. Three micrometers thick histological sections were stained with methylene blue for morphological studies. The sections were observed and photographed under a DM LS (Leica) light microscope coupled to a DFC420 camera (Leica) and Leica Application Suite version 3.1.0 imaging software.

### Transmission Electron Microscopy

Hemocytes from infected ticks (10^7^ conidia mL^−1^), ticks inoculated with Tween^®^80 and untreated ticks were collected 24 h after infection, removed from plasma (De Paulo et al., 2018), and fixed in 2% glutaraldehyde for 3 h at 4°C. The samples were then centrifuged at 500 × *g* for 3 min, the supernatant was discarded, the cells’ pellet was washed in 0.2 M Millonig buffer for 15 min, centrifuged at 500 × *g* for 3 min, and then post-fixed in 1% OsO_4_ at room temperature for 3 h. After that, pellets were washed in Millonig buffer, dehydrated in ascending acetone series (30, 50, 70, 90, and 100%), pre-embedded in Polybed 812 resin-acetone (1:1) overnight at room temperature, and embedded in pure resin at room temperature for 8 h. After resin polymerization (72 h) at 60°C, ultrathin sections were obtained, stained with uranyl acetate and lead citrate. The examination was performed in Jeol 100 CX II and LEO EM 906E transmission electron microscope.

### X-Ray Microanalysis of Cells Granules

The chemical composition of *R. microplus* granulocytes’ granules was determined by energy-dispersive X-ray spectrometer (TEM-EDS, H7650/Hitachi H-700 FA). Hemocytes from Tween-inoculated ticks and fungus-infected ticks (10^7^ conidia mL^−1^) were prepared as described for transmission electron microscopy and a quantitative characteristic of ions and maps of their distribution were obtained with a Hitachi H-8100 TEM microscope (Hitachi, Tokyo, Japan) equipped with energy-dispersive X-ray spectrometer (EDS, OXFORD INCA x-sight, Abingdon, Oxfordshire, United Kingdom).

### Statistical Analysis

Bioassay data were analyzed by one-way ANOVA followed by Newman-Keuls Multiple Comparison Test (*P* ≤ 0.05). Cell viability data were analyzed by one-way ANOVA followed by Tukey’s test for pair-wise comparisons (*P* ≤ 0.05). For analysis of hemocytes populations, multiple *t*-tests were used for pair-wise comparisons (*P* ≤ 0.05). All data were analyzed through GraphPad Prism version 5.00 for Windows (GraphPad Software, San Diego, CA, United States).

## Results

### Bioassay With Engorged Females

**FIGURE 1:**
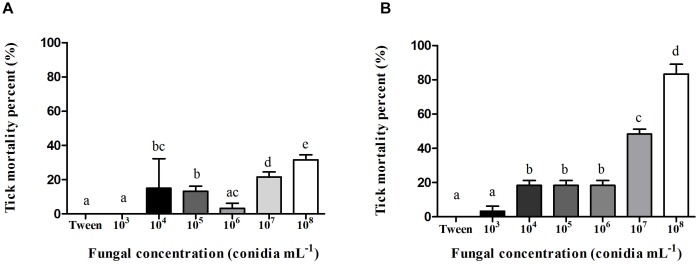
Mean percent (%) mortality and standard deviation of *Rhipicephalus microplus* adult females infected with *Metarhizium robertsii* ARSEF 2575 conidia in different concentrations (1.0 × 10^3^, 10^4^, 10^5^, 10^6^, 10^7^ and 10^8^ conidia mL^−1^). Each group had 20 tick females. Mortality percent was recorded **(A)** 24 h and **(B)** 48 h after fungal inoculation. Mean values (±) standard deviation followed for the same letter do not differ statistically by ANOVA test (*P* ≥ 0.05). The bioassay was conducted three times, on three different days, using new ticks and conidial preparations each day.

Conidia of *M. robertsii* isolate ARSEF 2575 used to infect adult females had 98% germination after incubation for 24 h at 27 ± 1°C and ≥80% RH. Females’ mortality percent is shown in Figure 1. In general, tick mortality was proportional to conidial concentration, (i.e., the higher the conidial concentration, the higher the tick mortality) (Figure 1A,B). Ticks infected with 10^7^ conidia mL^−1^ showed 21% ± 2.35 mortality 24 h after infection and 48% ± 2.35 mortality 48 h after infection, while ticks infected with 10^8^ conidia mL^−1^ had 31,66% ± 2.0 and 83,3% ± 4.71 mortality 24 and 48 h after infection, respectively.

### Cell Viability Test

**FIGURE 2:**
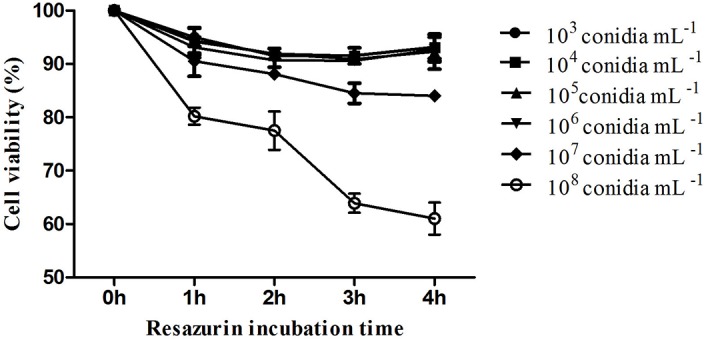
*In vitro* cytotoxic assay of *Metarhizium robertsii* ARSEF 2575 against *Rhipicephalus microplus* hemocytes using a resazurin-based assay (to evaluate cell viability). *R. microplus* hemocytes were exposed to different concentrations (1.0 × 10^3^, 10^4^, 10^5^, 10^6^, 10^7^, and 10^8^ conidia mL^−1^) of fungal suspensions. Twenty-four hours after exposure, resazurin was used for four different incubation times (1, 2, 3, and 4 h). ANOVA followed by Tukey’s test was used. Experiments were repeated with at least three independent biological samples.

**FIGURE 3:**
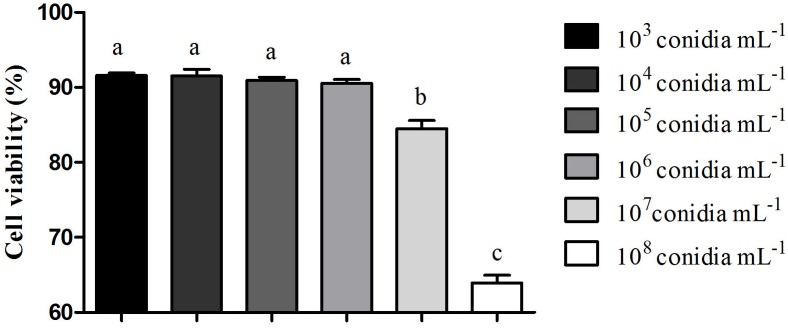
Effect of *Metarhizium robertsii* on the cellular proliferation of *Rhipicephalus microplus* hemocytes at 3 h incubation with resazurin. Mean values (±) standard deviation followed for the same letter do not differ statistically by ANOVA test (*P* ≥ 0.05). Experiments were repeated with at least three independent biological samples.

The kinetic curve shows hemocytes viability 24 h after fungal exposure in different times of incubation with resazurin (Figure 2). It was possible to observe that hemocytes viability was similar 3 and 4 h after incubation (Figure 2). *M. robertsii* ARSEF 2575 conidia were more toxic to *R. microplus* hemocytes at 10^7^ conidia mL^−1^ and 10^8^ conidia mL^−1^, reducing cell viability in 15, 53% ± 1.58 and 36.1% ± 1.48 24 h after the fungal addition, respectively (Figure 3). Despite lower fungal concentrations (10^3^, 10^4^, 10^5^, and 10^6^ conidia mL^−1^) can also influence cell viability, drastic reductions started at the higher concentrations (i.e., 10^7^ and 10^8^).

### Light Microscopy Analysis

**FIGURE 4:**
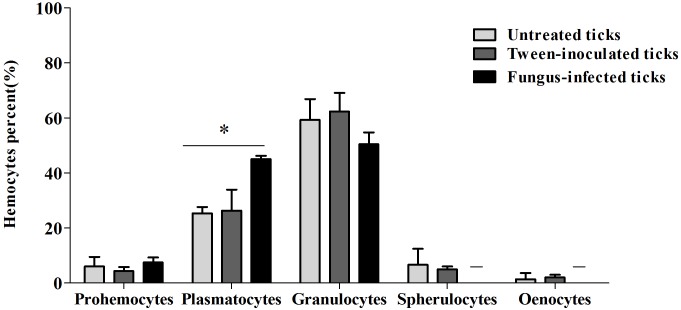
Mean percent and standard deviation of *Rhipicephalus microplus* hemocytes population (prohemocytes, granulocytes, plasmatocytes, spherulocytes and oenocytoids) of untreated tick females (ticks under physiological conditions), tween-inoculated ticks and fungus-infected ticks. *R. microplus* females were infected with *Metarhizium robertsii* ARSEF 2575. Mean values (±) standard deviation followed by ^∗^ differ statistically by multiple *t*-tests (*P* ≤ 0.05). A total of 100 cells were counted in each slide (five slides were analyzed for each group). Experiments were repeated with at least three independent biological samples.

**FIGURE 5:**
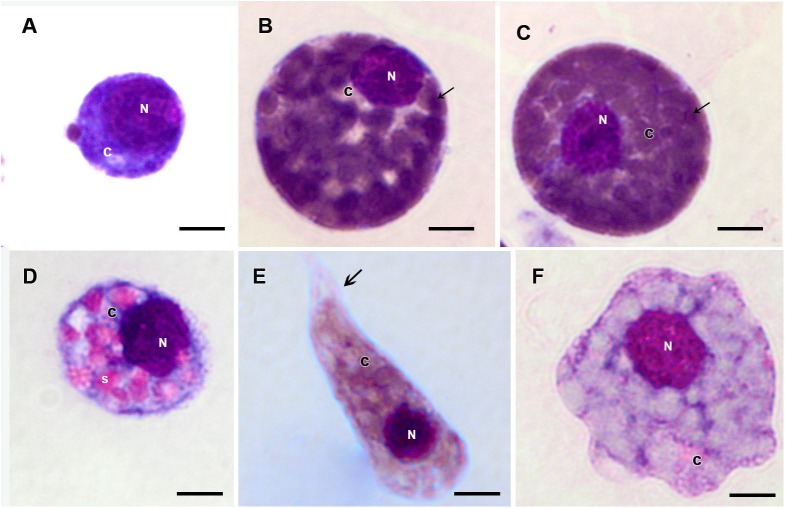
Hemocytes representation found in the hemolymph of *R. microplus* engorged females under physiological conditions (untreated ticks) after Giemsa staining. **(A)** Prohemocyte, **(B,C)** Granulocyte, **(D)** Spherulocyte, **(E)** Plasmatocyte and **(F)** Oenocyte. Nucleus (N), cytoplasm (c), granules (thin black arrow), spherules (s) and pseudopodia (thick black arrow). Bars = 5 μm.

**FIGURE 6:**
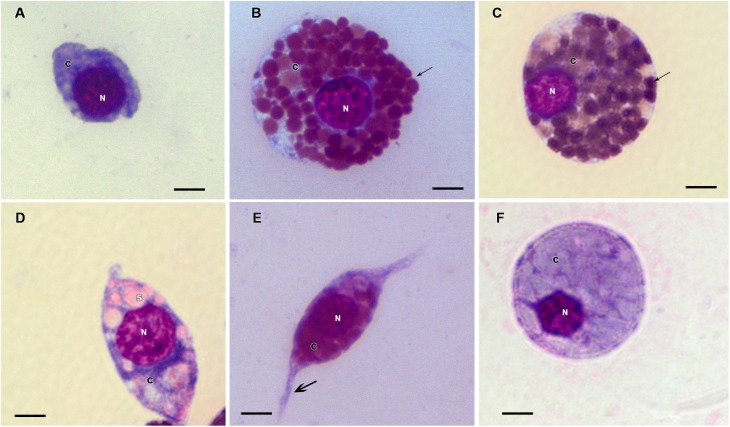
Hemocytes representation found in the hemolymph of *R. microplus* engorged females from tween-inoculated ticks after Giemsa staining. **(A)** Prohemocyte, **(B,C)** Granulocyte, **(D)** Spherulocyte, **(E)** Plasmatocyte and **(F)** Oenocyte. Nucleus (N), cytoplasm (c), granules (thin black arrow), spherules (s) and pseudopodia (thick black arrow). Bars = 5 μm.

**FIGURE 7:**
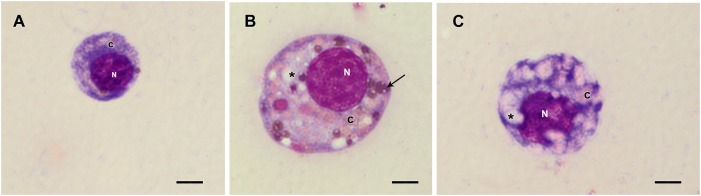
Hemocytes representation found in the hemolymph of *R. microplus* engorged females from fungus-infected ticks after Giemsa staining. **(A)** Prohemocyte, **(B)** Granulocyte and **(C)** Plasmatocyte. Nucleus (N), cytoplasm (c), vacuoles (^∗^) and granules (thin black arrow). Bars = 5 μm.

Prohemocytes, spherulocytes, granulocytes, plasmatocytes, and oenocytoids were observed in both engorged *R. microplus* females under physiological conditions and tween-inoculated ticks after Giemsa staining. The concentration of these hemocytes was the same for both groups (Figure 4). On the other hand, when engorged females were infected with *M. robertsii*, spherulocytes and oenocytoids were not observed. In addition, prohemocytes and granulocytes were present in the same amounts in controls and fungus-infected groups, only plasmatocytes amounts were statistically different between uninfected (negative control and tween-inoculated) and infected groups. All cells had the basophilic nucleus and the following characteristics: Prohemocytes (ranging from 8 to 10 μm) as relatively small cells, with large nucleus (Figure 5A, 6A). Granulocytes (measuring approximately 12 to 15 μm) as circular cells that were observed in two different forms: with a more central nucleus or eccentric nucleus (Figure 5B,C, 6B,C). Spherulocytes (measuring approximately 12 to 15 μm) had oval shape, with spherules in the cytoplasm (Figure 5D, 6D). Plasmatocytes (measuring approximately 20 μm) presented a varied shape, with the nucleus displaced of the central region of the cell; some plasmatocytes had pseudopodia (Figure 5E, 6E). Oenocytoids were larger-sized (approximately 20 μm) exhibiting the nucleus displaced of the central region of the cells (Figure 5F, 6F). Additionally, spherulocytes, plasmatocytes, and oenocytoids exhibited poorly Giemsa-stained areas (Figure 5D–F, 6D–F). Granulocytes from fungus-infected ticks showed fewer granules than hemocytes from untreated or tween-inoculated ticks. Prohemocytes were similar in uninfected or fungus-infected females (Figure 5A, 6A, 7A). The cells’ cytoplasm from infected ticks showed heterogeneity (Figure 7B) and also intense vacuolization (Figure 7C).

**FIGURE 8:**
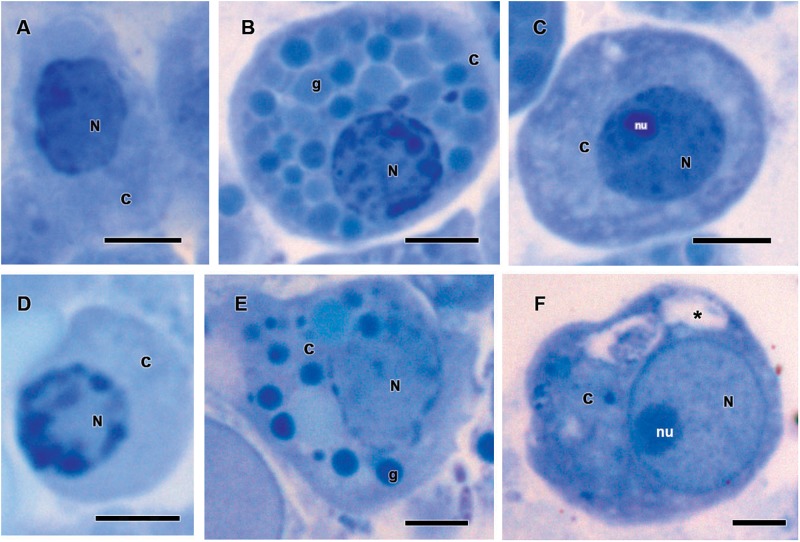
Hemocytes representation found in the hemolymph of *Rhipicephalus microplus* engorged females **(A–C)** inoculated with 0.1% Tween^®^80 aqueous solution (v/v) or **(D–F)** infected with *Metarhizium robertsii* after methylene blue staining. Prohemocytes **(A)** from tween-inoculated ticks and **(D)** from fungus-infected ticks; granulocytes **(B)** from tween-inoculated ticks and **(E)** from fungus-infected ticks; plasmatocytes from **(C)** tween-inoculated group and **(F)** from fungus-infected ticks. Nucleus (N), cytoplasm (c), nucleolus (nu), granule (g) and vacuoles (^∗^). Bars = 5 μm.

Historesin analysis was used to compare the hemocytes types [i.e., prohemocytes, granulocytes, and plasmatocytes (Figure 8)] observed in both fungus-infected and tween-inoculated ticks. After fungal infection, prohemocytes had the same morphology than the prohemocytes from tween-inoculated tick females (Figure 8A,D). Despite this, granulocytes apparently lost granules, since cells from infected females had fewer granules than the granulocytes from uninfected ticks (Figure 8B,E). Plasmatocytes of fungus-infected females also showed morphological changes, exhibiting vacuoles in their cytoplasm (Figure 8C,F).

### Transmission Electron Microscopy Analysis

**FIGURE 9:**
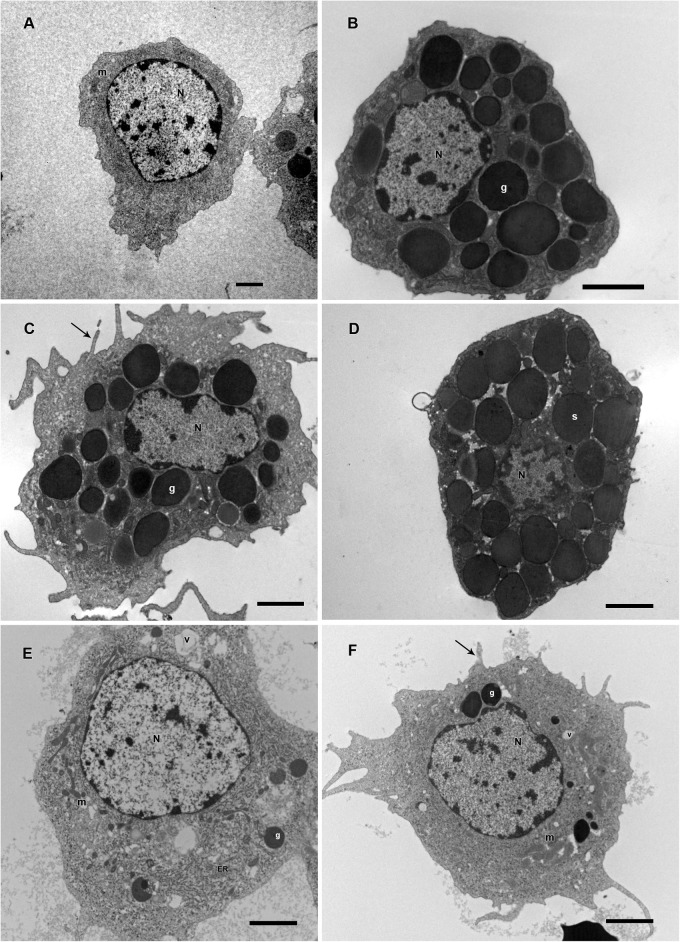
Electromicrographs of *R. microplus* hemocytes in normal physiological conditions (untreated-ticks). **(A)** Prohemocyte with nucleus (N) and mitochondria (m) in its cytoplasm. **(B,C)** Granulocytes with nucleus (N), electron dense granules (g), and plasma membrane projection (black arrow). **(D)** Spherulocyte with nucleus (N) and spherules (s) in the cytoplasm. **(E,F)** Plasmatocytes with nucleus (N), mitochondria (m), endoplasmic reticulum (ER), electron dense granules (g), vacuole (v), and plasma membrane projections (black arrow). Bars = 2 μm.

**FIGURE 10:**
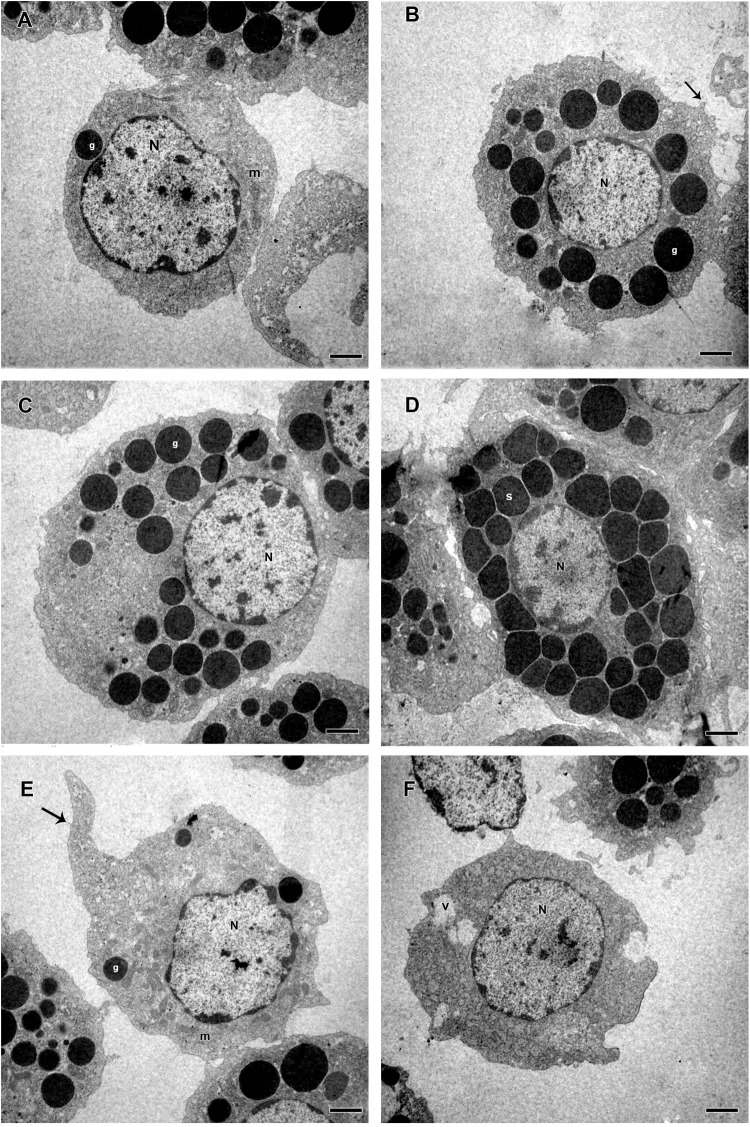
Electromicrographs of hemocytes from *R. microplus* engorged females inoculated with 0.1% Tween^®^80 aqueous solution (v/v). **(A)** Prohemocyte with nucleus (N), electron dense granule (g) and mitochondria (m) in its cytoplasm. **(B,C)** Granulocytes with nucleus (N), electron dense granules (g), and plasma membrane projection (black arrow). **(D)** Spherulocyte with nucleus (N) and spherules (s) in the cytoplasm. **(E,F)** Plasmatocytes with nucleus (N), mitochondria (m), electron dense granules (g), vacuole (v), and pseudopodia (black arrow). Bars = 2 μm.

Based on presence or absence of granules, cell shape, presence or absence of pseudopodia, nucleus location, endocytic activity, endoplasmic reticulum and homogeneity of the cytoplasm, *R. microplus* hemocytes were classified as follows: Prohemocytes: small cells with a large and central nucleus, none or few granules, and mitochondria (Figure 9A, 10A). Granulocytes: exhibited central or eccentric nuclei, cytoplasmic projections and lots of granules with different electron densities (Figure 9B,C, 10B,C). Spherulocytes: large cells with homogeneous electron densities spherules occupying virtually the entire cytoplasm (Figure 9D, 10D). Plasmatocytes: variable sized cells with no or few granular inclusions in the cytoplasm and pseudopodia (Figure 9E,F, 10E,F). Oenocytoids were not observed in TEM analysis. No differences were observed between untreated and tween-inoculated tick females, except granulocytes from ticks under physiological conditions apparently exhibited more plasma membrane projections (Figure 9C, 10C).

**FIGURE 11:**
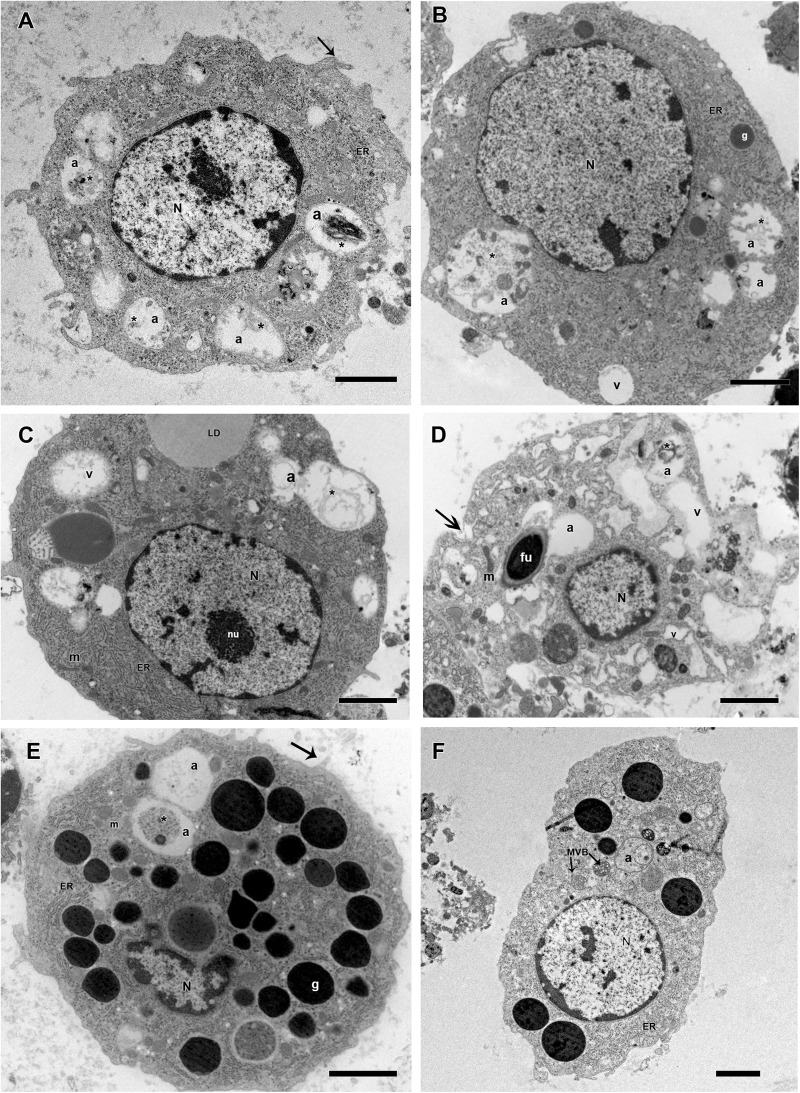
Electromicrographs of *R. microplus* hemocytes from fungus-infected ticks. **(A)** Plasmatocyte with nucleus (N), endoplasmic reticulum (ER), autophagosome (a) with material at different stages of degradation (^∗^) and plasma membrane projection (black arrow). **(B)** Plasmatocyte with nucleus (N), electron dense granules (g), vacuolization (v), endoplasmic reticulum (ER), autophagosome (a) with material at different stages of degradation (^∗^). **(C)** Plasmatocyte with nucleus (N), nucleolus (nu), mitochondria (m), endoplasmic reticulum (ER), presence of lipid droplets (LD) adjacent to low electron density vacuoles (v) and autophagosome (a) with material at different stages of degradation (^∗^). **(D)** Plasmatocyte with nucleus (N), double wall fungal conidia in the cytoplasm (fu), intense vacuolization (v), mitochondria (m), plasma membrane rupture (black arrow) and autophagosome (a) with material at different stages of degradation (^∗^). **(E)** Granulocyte with nucleus (N), electron dense granules (g), mitochondria (m), endoplasmic reticulum (ER), plasma membrane projection (black arrow) and autophagosome (a) with material at different stages of degradation (^∗^). **(F)** Granulocyte with nucleus (N), endoplasmic reticulum (ER), autophagosome (a) and multivesicular bodies (MVB). Bars = 2 μm.

Some morphological differences were observed in hemocytes of *R. microplus* ticks after fungal infection. Twenty-four hours after infection, prohemocytes, spherulocytes and oenocytoids could not be observed, only granulocytes and plasmatocytes. Plasmatocytes had intense cytoplasmic vacuolization and double wall fungal conidia in the cytoplasm (Figure 11A–D), suggesting phagocytosis of conidia. In addition, these cells displayed autophagosome with material at different stages of degradation, plasma membrane rupture, suggesting a necrosis process, healthy mitochondria, nucleus with normal appearance and an increased endoplasmic reticulum. Granulocytes from fungus-infected ticks had fewer granules than granulocytes of uninfected ticks, also exhibited autophagosomes with material at different stages of degradation and the presence of multivesicular bodies (Figure 11E,F). Hemocytes from fungus-infected tick females had nuclei showing extensive regions of euchromatin, several cytoplasmic vacuoles with different electron densities, subcellular material in degradation stage or already degraded, and the presence of autophagosome or multivesicular bodies, suggesting an autophagy process (Figure 11).

### Characterization of Cells Granules by Microanalysis

**FIGURE 12:**
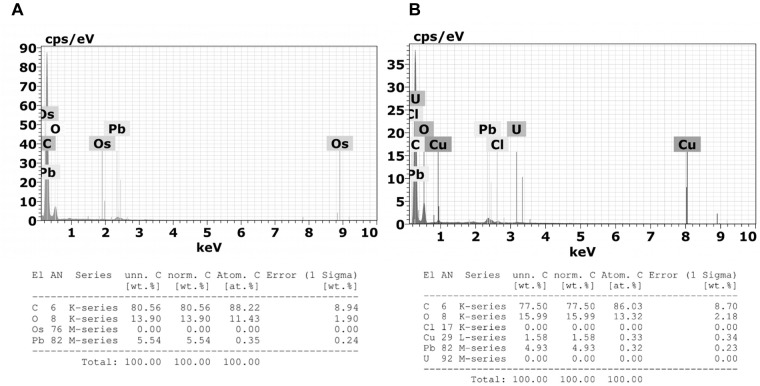
Emission spectra obtained by Energy Dispersive X-Ray Spectroscopy (X-EDS) in TEM performed on 3 μm sections of **(A)** granulocytes from tween-inoculated ticks and **(B)** granulocytes from fungus-infected ticks. Spectra obtained focusing the incident electron beam on the granulocyte electron-dense granules of which characteristic energies of non-X-ray emission are evident. Multiple X-EDS analyses have been performed in each group; the ones that exhibited a pattern in common with the rest of the acquired spectra are shown. It is also possible to observe the presence of copper (Cu), a component of the TEM supporting grid; osmium (Os), used as fixative; lead (Pb), and uranium (U) that are used as staining.

Microanalysis demonstrated that *M. robertsii* ARSEF 2575 infection did not affect the composition of *R. microplus* granulocytes’ electron dense granules (Figure 12). Granules of granulocytes from tween-inoculated ticks and fungus-infected ticks were composed only by oxygen and carbon.

## Discussion

Some entomopathogenic fungi are known to be highly virulent to ticks, as well as to different species of insects (Roberts and St Leger, 2004; Wang and St Leger, 2006; Leemon and Jonsson, 2008; Samish et al., 2014; Perinotto et al., 2017). Interestingly, entomopathogenic fungi have a variety of mechanisms that can neutralize the host defenses, such as the production of secondary metabolites able to suppress the insect immune system (Dubovskiy et al., 2013). This suppression of immune reactions is one of the main mechanisms governing the outcome of relations between a host and an invader. Furthermore, *Metarhizium* spreads in nutrient-rich hemocoel through immunological evasion and adaptation to osmotic stress (Wang and St Leger, 2006; Huang et al., 2015). Here, *M. robertsii* ARSEF 2575 was virulent to *R. microplus* engorged females after fungal inoculation. Several factors are involved in the virulence of a fungal isolate to arthropods, especially ticks that are much less susceptible than insects (Rot et al., 2013; Erler and Ates, 2015; Mohammadyani et al., 2016; Alves et al., 2017; Fischhoff et al., 2017). Distinct fungal virulence to different populations of the same host (Perinotto et al., 2012), host species, life stage of the host (i.e., some species and stages are more susceptible than others), and the quantity of fungal propagules applied to the arthropod pest (Alden et al., 2001; Ment et al., 2012) are some of these factors. Analyzing the results of the present study, the highest fungal concentration (i.e., 1.0 × 10^8^ conidia mL^−1^, the one more frequently used for tick control) in the longest time of infection (i.e., 48 h) yielded the best tick mortality rate; while the lowest fungal concentrations (i.e., 1.0 × 10^4^, 10^5^, and 10^6^ conidia mL^−1^) resulted in similar mortality rates. Despite the longer time of infection, greater the negative effects for the host. The mortality rates of ticks 48 h after fungal inoculation in the higher doses, prevented the hemolymph collection since hemolymph collected from near-to-death or dead females is not colorless but red, suggesting midgut disruption and fungal colonization of organs such as ovaries (Liu et al., 2009; Ment et al., 2012; Sanchez-Roblero et al., 2012; De Paulo et al., 2018). The same happens with the hemolymph collected 24 h after inoculation with the highest fungal concentration (1.0 × 10^8^ conidia mL^−1^). Accordingly, the ultrastructural and cytotoxic analyses of hemocytes from fungus-infected ticks were performed with hemolymph collected 24 h after fungal inoculation after inoculation with 1.0 × 10^7^ conidia mL^−1^.

During the fungal infectious process, the arthropod can fight the pathogen through humoral and cellular immunity; the last one is mediated by hemocytes (Sterba et al., 2011). The present study demonstrated that the entomopathogen *M. robertsii* negatively affected *R. microplus* tick hemocytes, possibly causing cell death. The kinetic curve of the hemocytes viability test 24 h after exposure to *M. robertsii* showed that the highest resazurin activation (i.e., the lowest hemocytes viability) occurred with 3 h of incubation, after this time, activity remained stable. There are no studies involving resazurin and tick hemocytes, or even evaluating entomopathogenic fungal toxicity to these cells. Most studies with arthropod hemocytes are focused on MTT [3-(4,5-dimethylthiazol-2-yl)-2,5-diphenyl tetrazolium bromide] (Jose et al., 2011) and assays performed over heavy metal toxicity (Katsumiti et al., 2014, 2015; Minguez et al., 2014). Analyze the cells’ viability is crucial for toxicity tests and MTT is widely used, however, it has lower sensibility and needs a higher incubation time in comparison to resazurin, once MTT measures the mitochondrial dehydrogenate (Mosmann, 1983). In addition, resazurin test is easy to be performed because it does not need culture media removal or organic solvents (Mosmann, 1983; Xu et al., 2015).

Studies about *R. microplus* cellular morphology and ultrastructure are scarce; for this reason, hemocytes identification through light and transmission microscopy was based on other tick species and other arthropods (Carneiro and Daemon, 1997; Borovicková and Hypsa, 2005; Habeeb and El-Hag, 2008; Feitosa et al., 2015, 2018). Silva et al. (2006) studies with light microscopy reported six hemocytes types in *R. microplus* hemolymph described as prohemocytes, granulocytes, plasmatocytes, spherulocytes, adipohemocytes, and oenocytoids. The last two were found to be less abundant. In the present study, only five cell types were observed: prohemocytes, granulocytes with different morphologies, plasmatocytes, spherulocytes, and oenocytoids, also diverging from studies with *R. sanguineus*, where adipohemocytes were also reported (Feitosa et al., 2015). After fungal infection, it was possible to observe that prohemocytes morphology was similar to prohemocytes from uninfected *R. microplus* ticks, unlike granulocytes and plasmatocytes. Granulocytes from fungus-infected ticks apparently lost granules and plasmatocytes of infected females showed cytoplasm vacuolization. The morphological alterations of plasmatocytes suggested the phagocytic activity and also may indicate a process to cell death, already reported by Sharma et al. (2008) and Shaurub et al. (2014). Some cells from untreated or treated ticks exhibited poorly stained areas (Figure 5–7), what is suggested to be a result of the differences between intracellular components. Accordingly, further studies are needed to characterize those areas for a better understanding of the organelles present in these cells.

Electron microscopy is very important for hemocytes characterization. Despite this, until the present date, the ultrastructural description of *R. microplus* hemocytes was not available in the literature. Accordingly, the morphological descriptions in the present study were compared with other tick species (Borovicková and Hypsa, 2005; Habeeb and El-Hag, 2008; Feitosa et al., 2015, 2018). Here, prohemocytes had similar morphology to those described by Habeeb and El-Hag (2008) and Feitosa et al. (2015) for other tick species. In the ultrastructure analysis, these cells showed ribosomes and mitochondria, but little endoplasmic reticulum and Golgi. The similar morphological characteristics of this hemocyte reported by this and other studies may be related to its function, since the prohemocyte is considered the precursor of other hemocytes (Lavine and Strand, 2002; Kavanagh and Reeves, 2004). Nevertheless, the origin of the other hemocytes is still unclear (Nation, 2016).

Plasmatocytes were abundant, polymorphic, had either central or eccentric nuclei, vesicles with different sizes, small vacuoles and mitochondria (Sonenshine and Roe, 2014). However, in the present study, cytoplasmic and pseudopodia projections, and an agranular or slightly granular cytoplasm were observed, in contrast to the findings of Feitosa et al. (2015) that described plasmatocytes with only small granulations. This and other studies suggested that this cell type is strongly involved in the immune response, through the removal of apoptotic cells, but mainly due to phagocytosis and encapsulation of pathogens, analogous to the actions of monocytes in vertebrates (Tojo et al., 2000; Lavine and Strand, 2002; Jiang et al., 2009; Honti et al., 2014).

Insect granulocytes are generally elliptic cells showing protease activity with acid phosphatase in the lysosomal compartments (Nation, 2016). For other tick species such as *Ornithodoros moubata* and *Ixodes ricinus*, granulocytes were distinguished in two types, based on the electron density and maturation of their granules (Borovicková and Hypsa, 2005; Sonenshine and Roe, 2014), while for *R. sanguineus* differentiation of granulocytes types was described based on cell maturation (Feitosa et al., 2015). In the present study, these cells had granules with different sizes, different electron densities and also diverse nucleus position in the cytoplasm. Despite this, we did not distinguish these cells in different types, since it was necessary to investigate the granules’ composition before staggering additional classifications.

Spherulocytes are less abundant cells in the hemolymph, however, in *R. sanguineus* they appear to be more abundant (Carneiro and Daemon, 1997; Feitosa et al., 2015). In the present study, these cells present spherules in the cytoplasm and less organelles corroborating with other studies with insects (Brayner et al., 2005) and tick (Sonenshine and Hynes, 2008). In insects, this cell type is suggested to be involved with tissue renewal, transport of substances such as hormones and even production of some hemolymph proteins (Negreiro et al., 2004).

Adipohemocytes are cells found in insects and some tick species with varying sized and shape lipid droplets, throughout the entire cytoplasm, some mitochondria, and eccentric nucleus (Cunha et al., 2009). In *Aedes aegypti*, this cell type is considered the second most abundant and may have some granules (Hillyer et al., 2003). In ticks, a study with *R. sanguineus* reported this cellular type was rarely found in the hemolymph (Feitosa et al., 2015). In the present study, this cell type was not observed, endorsing other studies with ticks (Borovicková and Hypsa, 2005; Habeeb and El-Hag, 2008). The absence of these cells in the present study is suggested to reflect its low abundance or absence in *R. microplus* hemolymph, or a misclassification since these cells are microscopically identical fat body cells or can even be mistaken by plasmatocytes with lipid droplets (Gupta, 1985; Hillyer et al., 2003; Nation, 2016).

Oenocytoids are cells the exhibit varied size and shape, without pseudopodia, and usually with an eccentric nucleus (Gupta, 1985). They are non-phagocytic cells but may be involved in the encapsulation process (Eggenberger et al., 1990). At light microscopy these cells show homogeneous cytoplasm or small refractory granulations (Carneiro and Daemon, 1997). Transmission microscopy revels few organelles, but some mitochondria and electron luscent vesicles may be present (Giulianini et al., 2003). In ticks, this hemocyte type is not abundant (Silva et al., 2006; Feitosa et al., 2015). In the present study few oenocytoids were detected and only at light microscopy. It is suggested that the low abundance of these cells is due to its easy disruption associated with calcium mobilization that activates protein kinase C and open calcium channels, inducing increased intracellular osmotic pressure, causing cell disruption (Shrestha et al., 2015).

Hemocytes have several functions, featuring protection against pathogens, production and secretion of peroxidases, encapsulation and phagocytosis (Nation, 2016). The interactions between entomopathogenic fungi and tick hemocytes are not well elucidated, so the morphological characterization of these cells and the changes caused by the infectious process can help to investigate the lower susceptibility that these arthropods have toward entomopathogenic agents.

Granulocytes from fungus-infected ticks showed apparently fewer granules and some cells also exhibited vacuolization. These cells are guided to initiate, mediate and terminate the encapsulation process, forming a monolayer on the foreign agent (Sonenshine and Hynes, 2008). Despite this, in the present study, encapsulation processes of *M. robertsii* conidia by granulocytes were not observed, possibly due to the infection stage here analyzed (24 h after infection), considered advanced.

Plasmatocytes from fungus-infected ticks exhibited cytoplasmic vacuolization and double wall fungal conidia in the cytoplasm, suggesting a process of conidia phagocytosis. Vacuolization in plasmatocytes was also observed in other arthropod species after pathogens challenging (Sharma et al., 2008; Shaurub et al., 2014). In addition, in the present study, cells from tick-infected ticks lost its filopodia, endorsing Ghasemi et al. (2013) reports, and exhibited an increased endoplasmic reticulum. The endoplasmic reticulum is a membranous network which is responsible for protein biosynthesis, for example, and also acts as calcium storage (Berridge et al., 2003). For this reason, endoplasmic reticulum has significant importance in cellular stimuli, nutrient availability or redox status. When one cell has abundance of this organelle, it means this cell is trying to produce and secrete more protein seeking to control adversities in the intracellular media (Zhang and Kaufman, 2008). Nevertheless, further studies are needed to investigate the complete process of phagocytosis or fungal internalization and the resulting autophagy hemocytes.

Non-visualization of other cell types such as prohemocytes, spherulocytes and oenocytoids in the tick hemolymph after fungal infection is suggested to reflect the reduction in the concentration of these cell types considering that these cells do not have specific functions in the cellular immune response, and, for that reason, they are supposed to be more vulnerable to the pathogenic infection (Lavine and Strand, 2002; Cunha et al., 2009). Prohemocytes may also be in a lower concentration because these cells may have differentiated into granulocytes and plasmatocytes (Feitosa et al., 2015). Additionally, secondary metabolites produced by *Metarhizium* can cause hemocytes apoptosis reducing the number of circulating hemocytes in the hemolymph (Wiegand et al., 2000).

The encapsulation of pathogens or foreign bodies is one of the most common defense processes in arthropods succeeding to encompass larger targets such as nematodes (Bronskill, 1962; Peters and Ehlers, 1997; Mastore et al., 2015). In insects, granulocytes are responsible for this process, in which several layers of the cells surround the pathogen, forming a capsule and inactivating it by the production of free radicals (Nappi and Ottaviani, 2000). In ticks, the process remains evident in plasmatocytes (Kopácek et al., 2000). In the present study, neither encapsulation nor nodulation processes were observed.

The fungal parasitism in arthropod cells has been reported for the fungus *Microsporidium*, which are eukaryotes, obligate parasites of several animal species and was also considered an entomopathogenic fungus (Polar et al., 2008). A large number of pathogens are able to survive in host cells, such as *Histoplasma capsulatum* fungus that can be phagocytosed by human alveolar macrophages and still is capable of multiplying itself within the cell (Newman et al., 2006). Supposedly, intracellular colonization of the fungus support itself, allowing some type of latency with a negative impact for the arthropod host health, although some factors as the nutritional stress can cause a rapid fungal growth, leading to host desiccation and arthropod death (Kurtti and Keyhani, 2008).

Exposure of insect hemocytes to pathogens such as viruses, bacteria, protozoa, and other microorganisms can trigger a cell response to the infection (Nation, 2016). Cells populations increase in hemolymph as a feedback to the infection process until the pathogens are eliminated. Nevertheless, when microorganisms are highly pathogenic, cells will be leaning to start autophagy processes. Additionally, the autophagic pathway seems to play an important role during the pathogen-host cell interaction, favoring or hindering the infection. Autophagy refers to the mechanism of degradation of cytoplasmic components through the lysosomal route, selectively or non-selectively. This process occurs physiologically throughout the cell cycle, but it is exacerbated under stress conditions such as nutritional deprivation. Autophagy promotes cell cycle modulation, growth, antigenic presentation, cytokine production, and degradation of intracellular pathogens in certain cell types (Munz, 2009). The imbalance of the autophagic pathway (increase or decrease) can lead to cell death (Kroemer and Levine, 2008). In the present study, an autophagosome was observed in hemocytes from fungus-infected ticks, suggesting an autophagic processes.

Previous studies involving autophagy were limited to its role in non-selective recycling of intracellular material to the lysosome, in response to starvation (Mizushima et al., 2004; Kroemer et al., 2010). Nevertheless, autophagy is now recognized as a process with several specialized functions, including elimination of large endogenous material in a selective way, such as mitophagy and xenophagy. Autophagosomes are identified by multiple assays, including fluorescent dyes and TEM ultrastructural analysis (Klionsky et al., 2012). Studies with *Drosophila melanogaster* and *Caenorhabditis elegans* demonstrated the contribution of autophagy to innate immune response, mainly when adaptive immunity is absent (Benjamin et al., 2013; Yin et al., 2016; Zimmermann et al., 2016; Kmiec et al., 2017). In *D. melanogaster*, autophagy is promoted by host pattern recognition receptors (PRRs), whereas in *C. elegans*, xenophagy is used to target *Microsporidia* (Bakowski et al., 2014). Recent studies started to unveil the involvement of autophagy with host tolerance rather than resistance to infection caused by extracellular pathogens (Kuo et al., 2018). Nevertheless, more investigation is required to elucidate why ticks are more tolerant to entomopathogenic fungi than insects, focusing especially in autophagy studies, since ticks have short life cycle.

The present study demonstrated the first evaluation of *R. microplus* hemocytes using transmission electron microscopy and elucidated some aspects associated to this tick’s cellular immune response to entomopathogenic fungi through cytotoxicity, TEM, and bioassay analysis, seeking to understand this host’s higher tolerance to entomopathogenic fungi in comparison to insects. Our study unveiled important observations necessary for the comprehension of tick physiology and tick pathology, supporting the progress of new strategies for the biological control of ticks.

## Data Availability

The raw data supporting the conclusions of this manuscript will be made available by the authors, without undue reservation, to any qualified researcher.

## Ethics Statement

This study was carried out in accordance with the project approved by the Ethics Committee for the Use of Animals of the Veterinary Institute, Federal Rural University of Rio de Janeiro (CEUA UFRRJ n°. 037/2014).

## Author Contributions

JF, CC-R, PG, IA, RM-B, and VB conceived and designed the study. JF, CC-R, and RB performed the experiments. DS-M helped with histological analyses. DS-M and RM-B helped with transmission electron microscopy analyses. JF and PG analyzed the data and drafted the manuscript. All authors read and approved the final manuscript.

## Conflict of Interest Statement

The authors declare that the research was conducted in the absence of any commercial or financial relationships that could be construed as a potential conflict of interest.
